# Methylation, Transcription, and Rearrangements of Transposable Elements in Synthetic Allopolyploids

**DOI:** 10.1155/2011/569826

**Published:** 2011-05-15

**Authors:** Beery Yaakov, Khalil Kashkush

**Affiliations:** Department of Life Sciences, Ben-Gurion University of the Negev, Beer-Sheva 84105, Israel

## Abstract

Transposable elements (TEs) constitute over 90% of the wheat genome. It was suggested that “genomic stress” such as hybridity or polyploidy might activate transposons. Intensive investigations of various polyploid systems revealed that allopolyploidization event is associated with widespread changes in genome structure, methylation, and expression involving low- and high-copy, coding and noncoding sequences. Massive demethylation and transcriptional activation of TEs were also observed in newly formed allopolyploids. Massive proliferation, however, was reported for very limited number of TE families in various polyploidy systems. The aim of this review is to summarize the accumulated data on genetic and epigenetic dynamics of TEs, particularly in synthetic allotetraploid and allohexaploid wheat species. In addition, the underlying mechanisms and the potential biological significance of TE dynamics following allopolyploidization are discussed.

## 1. Introduction

Some DNA sequences possess the unique ability to move from one place in the genome to another, these sequences are usually termed transposable elements (TEs). TEs makeup a large fraction of most eukaryotic genomes, particularly grasses, where they occupy up to 90% of the genome [1]. TEs are classified into two main groups, based on the intermediate molecule that mediates their movement: (1) RNA elements (retrotransposons or class 1 transposons) have RNA as their intermediate molecule; and (2) DNA elements (class 2 transposons) have DNA as their intermediate molecule [2]. 

TEs are considered “parasitic”, as the success of their reproduction is negatively correlated with the fitness of the host organism [3]. Some TEs have a marked preference for insertion within or near the vicinity of genes [4]. TE transposition can cause chromosome breakage, illegitimate recombination and genome rearrangement [3]. In addition, TEs can also affect gene expression if positioned into or near the gene [5–7]. In order to control their activity, TEs are mostly heavily methylated by the host, and as such, are associated with heterochromatin [3]. 

The bias of methylation toward repetitive DNA suggests that silencing of transposable elements is one of the primary roles of DNA methylation [8]. The *Arabidopsis* genome contains 24% methylated CG sites, 6.7% methylated CHG sites (H = A, C or T) and 1.7% methylated CHH sites [9]. The entire sequence of transposable elements is usually methylated in *Arabidopsis*, in all sequence contexts [8]. Considering that DNA demethylation or hypermethylation of transposable element sequences is associated with their activation or silencing, respectively. Usually TEs are hypermethylated compared to host genes in plants [10–12].

Allopolyploidy is a process where two genomes (being themselves polyploids or not) are brought together, usually by hybridization, into the same nucleus, followed by genome doubling. This new allopolyploid species is genetically isolated from its progenitors. The two genomes can be different species or different genera, and the resulting hybrid is sterile until genome doubling occurs. Allopolyploids typically have bivalent pairing, full fertility and disomic inheritance [13].

In 1970, Ohno proposed that evolution moves forward via whole genome duplication [14], an idea which is gaining momentum due to more sensitive sequence analysis used to investigate polyploidy [13]. Previous studies have shown that allopolyploidy can accelerate evolution in two ways: (1) rapid and reproducible genomic changes in the first generation of nascent polyploids, including elimination of DNA sequences [15–20], gene silencing [21–26], alteration of cytosine methylation [18, 27], and activation of genes and retrotransposons [5, 12, 28] and (2) sporadic genomic changes that occur during the lifetime of a polyploid species, which are not possible at the diploid level, such as diversification of homoeoalleles via mutations [29]. 

Synthetic wheat species (*Aegilops-Triticum* group) are used as a model to study the “smoking gun” of allopolyploid evolution (see review [13]). Newly synthesized wheat allopolyploids, which mimic natural polyploids, are produced from F1 hybrids treated with the mitotic inhibitor colchicine or by selfing spontaneously unreduced gametes of F1 hybrids [17]. *T. aestivum *is a hybridization between the tetraploid *T. turgidum *(genome BBAA, 2*n* = 4*x* = 28) and the diploid *Ae. tauschii *(genome DD, 2*n* = 2*x* = 14). The tetraploid *T. turgidum *is itself a hybridization of *T. urartu *(genome AA, 2*n* = 2*x* = 14) and an unknown genome BB diploid donor. The leading candidate progenitor of the BB genome is *Ae. speltoides *(genome SS, 2*n* = 2*x* = 14), as its genome is the closest to that of *T. turgidum* [30]. 

one of the major responses to an allopolyploidization event in wheat is elimination of DNA sequences: low-copy coding and noncoding sequences [17, 18, 31, 32] as well as repetitive sequences [19, 33–36]. In addition, changes in the wheat transcriptome as a result of allopolyploid were shown in wheat [25], yeast [37], maize [38], and synthetic and natural *Arabidopsis* allotetraploids ([23] and [24], resp.). 

In this paper, we review the accumulated data on TE dynamics following allopolyploidization in order to shed light on the possible mechanisms that are involved in TE regulation.

## 2. Allopolyploidy-Induced Transcriptional Activation of Retrotransposons

It is now evident that, under normal conditions, some retrotransposon promoters retain activity and initiate transcription of either the transposon itself or readout transcription to adjacent host DNA sequences [5, 39–41]. Our studies and others have shown that allopolyploidization might increase the steady-state level of expression of some transposons [5, 42]. In *Arabidopsis*, both DNA and RNA transposons displayed a higher transcriptional activity in synthetic allopolyploid hybrid compared with its autotetraploid parental lines [42]. For example, the *En-Spm*-like transposon, belonging to a novel family in *Arabidopsis* termed *Sunfish*, displayed higher transcriptional activity level in the synthetic allopolyploid hybrid, and this transcription was correlated with reduction in cytosine methylation of the element. 

In synthetic allotetraploid wheat, we have observed higher transcriptional activity of the LTR retrotransposon termed *WIS 2-1A* compared with its diploid parental lines [25]. Later, we have observed that this transcriptional activity leads to the production of readout transcripts toward adjacent host DNA sequences, a process that occurred in a genome-wide manner [5]. In many cases, these readout transcripts altered the expression of the adjacent genes based on their orientation: knocking down or knocking out the gene product if the readout transcript was in the antisense orientation relative to the orientation of the gene transcript; or overexpressing the gene if the readout transcript was in the sense orientation. 

Several studies reported specific cases, where a transposon insertion near a gene had influenced the expression of the gene, such as: an *Spm *insertion near the *a* locus and a *Mu *insertion near *hcf106* in maize (see [43, 44], resp.), and a *foldback-like* insertion near the *Drosophila* developmental *CG13617* gene [45]. However, the mechanisms by which this occurs are not well understood. In some cases, the reduction of the sense expression of the gene was correlated with the production of the antisense strand that initiated from the adjacent transposon promoter [5, 45]. This might indicate that posttranscriptional gene silencing might be responsible in silencing the adjacent genes. In addition, we have showed that this phenomenon might occur in a genome-wide manner in the first generations of newly formed wheat allopolyploid [5]. Whether this phenomenon is beneficial for genome stabilization of the emerging new allopolyploid species in nature remains a mystery. Future studies are required to investigate whether the genome-wide, high level readout transcription activity following allopolyploidization is temporal, namely, restricted to the first generation(s) of the newly formed allopolyploid. Our recent studies on tracking methylation changes around a retrotransposon in the first four generations of a newly formed wheat allopolyploid [46] may indicate that indeed this might be the case if the methylation of TEs is directly connected with readout transcription activity, as was shown in rice [41].

## 3. Allopolyploidy-Induced Massive Methylation Alterations near TEs

Epigenetic regulation of TEs, especially cytosine methylation, was shown to be relaxed following allopolyploidization, causing transcriptional activity of a TE promoter [5, 18, 25]. Thus, the alteration of methylation status following allopolyploidization was examined in *Arabidopsis *[27, 47], *Spartina* [48], and *Brassica *[49, 50] and surrounding TEs in wheat [18] and *Spartina* [51]. The methylation alterations are either hyper- or hypomethylation, depending on the sequence analyzed, and are reproducible.

**Figure 1:**
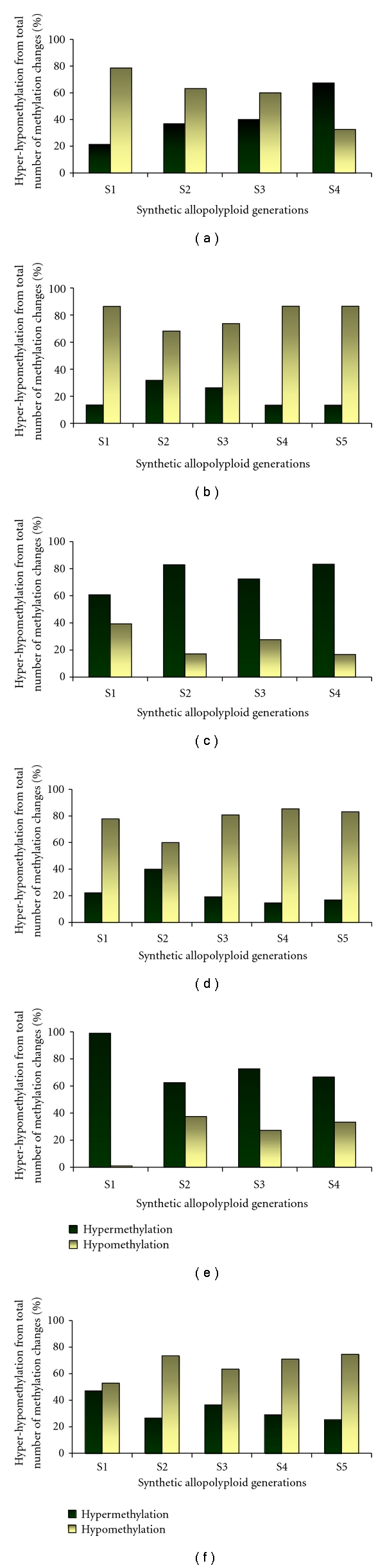
Type of changes in TMD patterns (hypomethylation versus hypermethylation) in the first four generations of the synthetic allohexaploid (S1–S4) and in the first five generations of the synthetic allotetraploid (S1–S5). (a) and (b) Corresponding to *Veju* elements in allohexaploid and allotetraploid, respectively; (c) and (e) corresponding to *Balduin* elements in allohexaploid and allotetraploid, respectively; (d) and (f) corresponding to *Thalos* elements in allohexaploid and allotetraploid, respectively.

In two recent studies, we have investigated the methylation of CCGG sites around several TE families. In one study [46], we have applied transposon methylation display (TMD) analysis (see [41]) on a terminal-repeat retrotransposon in miniature (TRIM), termed *Veju*, on *Triticum turgidum *ssp. *durum* (genome AABB) and *Aegilops tauschii *(genome DD), and the first four (S1–S4) generations of the derived allohexaploid. We estimated that about 55% of the CCGG sites flanking *Veju* elements showed altered TMD patterns (compared to the TMD patterns in the parental lines) in the first four generations (S1–S4) of the newly formed allohexaploid. In most cases, *Veju* sites were hypomethylated in the first generation (S1) of the newly formed allohexaploid, while hypermethylation was predominant in S4 generation (Figure 1(a)). This might be an indication for a reduction in* Veju* activity after the third generation of the synthetic allohexaploid. A similar pattern of hypomethylation of *Veju* elements was observed also in the first three generations (S1–S3) of synthetic allotetraploid that was derived from a cross between *Ae. sharonensis *(genome S^1^S^1^) and *T. monococcum *ssp. *aegilopoides *(genome A^m^A^m^). However, unlike in the synthetic allohexaploid, *Veju* elements remained hypomethylated also in the fourth generation of the synthetic allotetraploid (Figure 1(b)). 

As opposed to retrotransposons, class 2 elements (DNA elements) displayed completely different patterns in synthetic allohexaploid versus synthetic allotetraploid (see examples in Figures 1(c) and 1(d) versus 1(e) and 1(f), resp.). While the three investigated elements (*Balduin* (belonging to the *CACTA* superfamily), *Apollo *(belonging to the *MuDR/Foldback* superfamily) and* Thalos* (a *stowaway*-like* MITE* belonging to the Tc1/*mariner *superfamily)) underwent massive hypermethylation in the first four generations of the synthetic allohexaploid (see [52] and see data for *Balduin* and *Thalos* in Figures 1(c) and 1(d)), they underwent hypomethylation in the first four generations of the synthetic allotetraploid (Figures 1(e) and 1(f)). The massive hypermethylation of class 2 elements in the synthetic allohexaploid might be connected to the lack of transpositional activity [52], while some transpositional activity was documented in the synthetic allotetraploid (data not shown). This activity might be correlated to the hypomethylation of the elements in the synthetic allotetraploid.

**Figure 2:**
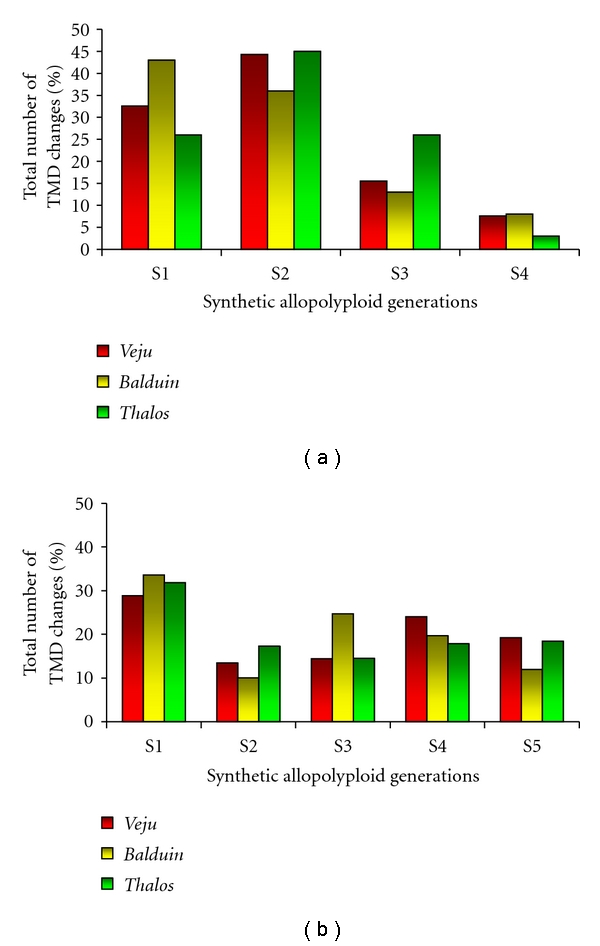
Timing of changes in TMD patterns in the first four generations of the synthetic allohexaploid (S1–S4) and in the first five generations of the synthetic allotetraploid (S1–S5). The % in each generation represents the fraction of changes at this particular generation, relative to the global final level of changes observed. For the three elements (*Veju *(red bars), *Balduin* (yellow bars), *and Thalos* (green bars)), the level of changes per generation from the total number of TMD bands subjected to methylation changes are shown in the synthetic allohexaploid (a) and in the synthetic allotetraploid (b).

Another important difference between the allohexaploid and the allotetraploid systems was the timing of changes in TMD patterns. Most of the changes (over 70% for the three elements: *Veju*, *Balduin,* and *Thalos*) in the synthetic allohexaploid occurred in the first two generations (levels of changes are maintained in the S2 and are even higher than in S1 for two TEs out of the three) (see [46, 52] and see Figure 2(a)), while ~30% (of the three elements) of the changes occurred in the first generation (S1) of the synthetic allotetraploid, followed by nearly a similar rate (~17% on average) in the subsequent generations (Figure 2(b)), in addition, in hexaploid system.

## 4. Allopolyploidy-Induced Genomic Rearrangements including TE-Containing Sequences

Early evidence for rearrangements following allopolyploidization came from an analysis of the polymorphism in the ribosomal DNA spacer of *Triticum *and *Aegilops *[53]. This was followed by a study revealing elimination of low-copy chromosome- and genome specific sequences in newly formed allohexaploid wheat, where it was suggested that these eliminations contribute to the disomic inheritance, essential to nascent allopolyploids [31]. Liu et al. later published two studies which show nonrandom genome specific sequence elimination and group-specific rapid genomic changes following polyploidization (see [15, 16], resp.). Shaked et al. [18] and Ozkan et al. [17] continued to show evidence of low-copy sequence elimination in allopolyploid wheat, using AFLP and Southern blots, respectively. These studies also showed the timing of elimination to be dependent on the plant species crossed and whether the elimination was chromosome or genome specific. Kashkush et al. [25] reported gene loss in newly formed wheat allotetraploid. A tandem DNA repetitive sequence was also shown to be eliminated, using *FISH* in allopolyploid wheat [19]. Up to ~70–90% of the copies were eliminated by the second to third selfed generations [19].

The prevalence of transposable elements and their inherent sequence similarity makes them a prime target for illegitimate and nonhomologous recombination. In tobacco, parent-specific retrovirus repeats and satellite repeats showed a partial or complete rapid elimination following allotetraploidization [20], and transposable elements have also been shown to undergo rearrangements following allopolyploidization in spartina and tobacco (see [51, 54], resp.). Bento et al. [55] detected genomic rearrangements in genes of the *triticale *polyploidy, as well as in retrotransposons.

Our recent data indicate that deletion of retrotransposon-containing sequences occur in the first three generations (S1–S3) of a synthetic allohexaploid, while no deletion events were detected in S4 [46]. This may indicate that deletion of DNA sequences following allopolyploidization is a rapid process, occurring in the first generations. This prediction was also reported by Ozkan et al. [17], where they proposed that deletion of low-copy sequences in newly formed wheat allopolyploids was completed in the S3 generation. 

In addition, we clearly showed that a change in the methylation status (usually hypomethylation) in the S1 generation was followed by deletion in the S2 generation [46]. These data plainly show the correlation between methylation and postallopolyploidization rearrangements that occur via a mechanism that is yet to be identified.

## 5. Transpositional Activity of TEs following Allopolyploidization

Despite the altered methylation status and transcriptional activation of TEs following allopolyploidization, there were very limited reports on transpositional activity of transposons. Madlung et al. [42] showed both methylation alterations along with limited transpositional activation of a *Sunfish* transposon in polyploidy *Arabidopsis*. Petit et al. [54] showed an increase in the copy number of a *Tnt1* retrotransposon in allotetraploid tobacco. No transposition bursts were reported in *Spartina* [51] and in wheat [5, 52, 56]. These reports indicate that the transpositional activity of TEs following allopolyploidization might be restricted to specific TE families [57]. 

Recently, we found that the immense loss of *Veju* sequences in the first generation after genome doubling is probably followed by retrotransposition in subsequent generations, a process that causes new insertions to accumulate in allohexaploids [46]. We also showed that these new insertions were rapidly targeted for methylation [46]. 

A comparison of the genomic distribution of *Veju* in the fifth (S5) generation of the synthetic allohexaploid and in the ~10,000-year-old natural allohexaploid revealed similar quantities (copy number) of *Veju* sequences. This might indicate that most rearrangements (deletion followed by accumulation) might occur in the earliest generations of the nascent allopolyploid rather than on an evolutionary scale. This explains the data published by Charles et al. [58], according to which allopolyploidization neither enhanced nor repressed retrotranspositions when tested on an evolutionary time scale.

## 6. Underlying Mechanism of TE Rearrangements

Although no mechanism for DNA elimination is currently accepted, Devos et al. [59] and Bennetzen et al. [60] suggested that illegitimate recombination, and to a lesser degree unequal homologous recombination, may be involved in the variation of genome sizes among angiosperms and the mechanism counteracting genome expansion by allopolyploidization and retrotransposon amplification. An analysis of a BAC sequence in allotetraploid cotton and its diploid progenitors revealed small deletions and illegitimate recombination following allopolyploidization [61]. In wheat, genomic sequences from a diploid and tetraploid species were compared [62], and they showed DNA rearrangements in repeat-rich regions, which might be attributed to illegitimate recombination. Chantret et al. [63] examined the *Hardness* locus in wheat, which underwent deletions following allopolyploidization, the sequences of the rearrangements and rearrangement breakpoints, and sequence motifs, suggesting illegitimate recombination as the underlying mechanism.

It is very important to mention that yet we do not know whether the insertional activity, for example, of *Veju *elements in synthetic allopolyploids, was caused by typical retrotransposition or, alternatively, by illegitimate integration. Experiments are on the way to characterize the new *Veju*-insertion loci and to identify target site duplications (TSDs), then check for empty sites in the parental lines by site-specific PCR.

## 7. Concluding Remarks

Transposable elements can act as “hot spots” that attract illegitimate rearrangements. The molecular mechanism of which hypomethylated elements undergo deletion remains unknown. This might be explained by hypomethylation conferring an open chromatin structure to the TE sequences, which exposes these demethylated elements to be targeted for deletion by the host. In addition, small RNAs might also have a major role in this process. There is evidence that small RNA corresponding to *Veju* elements might play a prominent role in *Veju* methylation in the newly formed wheat allohexaploid (Avi Levy, personal communication).

Methylation of new TE insertions can be understood as a defensive mechanism of the host from the deleterious transposon insertions. Investigating the scale of eliminated DNA sequences, including TE sequences, by identifying the deletion breakpoints will allow a better understanding of the mechanism(s) involved and of the nature of the connection between methylation and rearrangements, because it is believed that methylation of DNA usually occurs locally.

Many questions remained unanswered those include; (1) the biological role, if any, of the TE rearrangements following allopolyploidization; and (2) if indeed DNA rearrangements, following allopolyploidization, are a reprogrammed process as was suggested by Feldman and Levy [64]. Nevertheless, future studies should address these processes and their biological significance in nascent allopolyploid species.
